# A qualitative investigation of resilience and well‐being among medical physics residents

**DOI:** 10.1002/acm2.13554

**Published:** 2022-02-07

**Authors:** Kelly C Paradis, Kerry A Ryan, Spencer Schmid, Jean M Moran, Anna Laucis, Christina H Chapman, Terri Bott‐Kothari, Joann I Prisciandaro, Samantha Simiele, James M Balter, Martha M Matuszak, Vrinda Narayana, Reshma Jagsi

**Affiliations:** ^1^ Department of Radiation Oncology University of Michigan Ann Arbor Michigan USA; ^2^ Center for Bioethics and Social Science in Medicine University of Michigan Ann Arbor Michigan USA; ^3^ Department of Medical Physics Memorial Sloan Kettering Cancer Center New York City New York USA; ^4^ Department of Radiation Physics MD Anderson Cancer Center, University of Texas Houston Texas USA

**Keywords:** Residency training, resilience, well‐being

## Abstract

**Purpose:**

Medical physics residents (MPRs) will define and shape the future of physics in medicine. We sought to better understand the residency experience, as related to resilience and well‐being, through the lens of current MPRs and medical physicists (MPs) working with residents.

**Methods and materials:**

From February–May 2019, we conducted 32, 1‐h, confidential, semi‐structured interviews with MPs either currently enrolled in an accredited residency (*n* = 16) or currently employed by a department with an accredited residency (*n* = 16). Interviews centered on the topics of mentorship, work/life integration, and discrimination. Qualitative analysis methods were used to derive key themes from the interview transcripts.

**Results:**

With regard to the medical physics residency experience, four key themes emerged during qualitative analysis: the demanding nature of medical physics residencies, the negative impacts of residency on MPRs during training and beyond, strategies MPRs use to cope with residency stress, and the role of professional societies in addressing residency‐related change.

**Conclusions:**

Residency training is a stress‐inducing time in the path to becoming a board‐certified MP. By uncovering several sources of this stress, we have identified opportunities to support the resiliency and well‐being of MPs in training through recommendations by professional societies, programmatic changes, and interventions at the department and residency program director level for residency programs, as well as strategies that MPRs themselves can use to support well‐being on their career journey.

## 1 INTRODUCTION

Completion of a residency accredited by the Commission on Accreditation of Medical Physics Education Programs (CAMPEP) is required for American Board of Radiology certification in medical physics.^1^ In addition to the comprehensive training provided in preparation for becoming a safe and effective practicing medical physicist (MP), this requirement is a primary reason that formal residencies in the field of medical physics are highly sought‐after and competitive positions.

Medical physics residents (MPRs) play an essential role in the clinic, performing and participating in a myriad of clinical, educational, and research‐related projects and tasks. Correspondingly, residency program directors and participating faculty and staff MPs serve a pivotal role to ensure that each graduating resident has the required foundation of knowledge to transition safely into a clinical position. Residencies are time‐limited (ranging from 2–4 years) and the magnitude of the required didactic training material and associated clinical experience is considerable.^2^ This, combined with long and nonstandard working hours due to limited access to clinical equipment, the critical need for accuracy in patient‐related tasks, and relatively high workload (defined as “the overall cost incurred by a human operator to achieve a particular level of performance”),^3^ creates a challenging work environment. Although to our knowledge no studies have focused specifically on MPR‐related well‐being, in a 2019 study of peer support needs for MPs within the American Association of Physicists in Medicine (AAPM) by Johnson et al., 32.8% of participating MPs reported “frequent or constant burnout”,^4^ and a 2020 survey of 308 MPs within the European Society for Radiotherapy and Oncology found that 30% reported high scores on their burnout scale.^5^ We can extrapolate from observational studies within the physician workforce that these challenging work environments and high levels of burnout have the potential to risk patient safety, limit the talent pool of individuals choosing to enter and/or remain in the field, and have a devastating impact on the workforce.^6^, ^7^, ^8^, ^9^, ^10^, ^11^, ^12^, ^13^

In this study, we sought to understand the current medical physics residency experience as it relates to resident well‐being, and how resilience may play a role in successfully graduating from a residency program and moving on to a career as a board‐certified MP. The current report represents a sub‐section of a mixed‐methods study exploring work/life integration, mentorship, and discrimination within the field of medical physics. To our knowledge, this is the first qualitative study investigating the perceptions and experiences of the MPR workforce as it relates to resident resiliency and well‐being.

## 2 METHODS AND MATERIALS

This study was approved by the University of Michigan Institutional Review Board. A detailed description of the methods used in this study may be found in Paradis et al.^14^ Our study team consisted of an MP trained in qualitative methods (Kelly C Paradis), a radiation oncologist with a doctorate in social science (Reshma Jagsi), a senior research assistant/sociologist with experience as a qualitative interviewer, facilitator, and data analysis (Kerry A Ryan), an undergraduate research assistant trained in qualitative methods (Spencer Schmid), and additional MPs and radiation oncologists who provided contributions to study design and data interpretation (Jean M Moran, Anna Laucis, Christina H Chapman, Terri Bott‐Kothari, Joann I Prisciandaro, Samantha Simiele, James M Balter, Martha M Matuszak, and Vrinda Narayana). Briefly, from February to May of 2019, we recruited 32 practicing MPs and MPRs from across the US to participate in confidential, 1‐h, semi‐structured interviews on the topics of work/life integration, mentorship, and discrimination within the field of medical physics. Interviews occurred concurrently with recruitment efforts. Recruiting was performed by emailing the program directors of 96 CAMPEP‐accredited medical physics residency programs and asking the directors to distribute the invitations to the medical physics staff, faculty, and residents working at their institutions (international programs, non‐hospital‐affiliated programs, and the host institution were excluded; all other programs were contacted). There were 41 respondents to the initial invitation, all of whom were screened for the institution, job rank, gender, and other demographics (see Table 1). Nine respondents were not interviewed: five because we limited participation to no more than two per program, three because of gender or job rank cap (see below), and one was not from a CAMPEP‐accredited program. All respondents who were offered an interview ultimately participated in the interview process. The participants were purposefully sampled across gender (gender was self‐reported as either man or woman in this study) as well as between residents and staff/faculty physicists (where the distinction between staff and faculty is whether or not the participant had an academic appointment). Although invitations were sent to all eligible CAMPEP accredited programs (as described above), we did not purposefully sample by geographic location or race/ethnicity due to the initial estimated total sample size of 32 participants. All participants were employed within a department with an associated medical physics residency program, and all had roles that were primarily clinical. Participant confidentiality was protected by having a non‐MP conduct the interviews (Kerry A Ryan), transcribing the interview recordings, and adding redactions as necessary prior to any analysis taking place. Participants were provided with a $100 honorarium for taking part in the study.

**TABLE 1:** Interview participant demographic breakdown (total and by job rank)

	***n* (% or SD)**	**Resident** ***n* (% or SD)**	**Staff or faculty** ***n* (% or SD)**
Gender			
Woman	16 (50%)	8 (50%)	8 (50%)
Man	16 (50%)	8 (50%)	8 (50%)
Age, Mean (SD)	37.5 (7.4)	33.8 (5.5)	41.3 (6.9)
Years in practice (SD)	7.5 (6.6)	2.9 (2.7)	12.1 (6.0)
**Race/ethnicity (Select all that apply)**			
African American or Black	2 (6.3%)	1 (6.3%)	1 (6.3%)
American Indian or Alaska Native	2 (6.3%)	1 (6.3%)	1 (6.3%)
Asian American or Asian	5 (15.6%)	1 (6.3%)	4 (25.0%)
Hispanic or Latino	3 (9.4%)	2 (12.5%)	1 (6.3%)
Middle Eastern	2 (6.3%)	1 (6.3%)	1 (6.3%)
Multiracial	4 (12.5%)	3 (18.8%)	1 (6.3%)
Pacific Islander	0 (0%)	0 (0%)	0 (0%)
White or Caucasian	22 (68.8%)	14 (87.5%)	8 (50.0%)
Other	1 (3.1%)	0 (0.0%)	1 (3.1%)
**Job rank**		N/A	N/A
Faculty	12 (37.5%)		
Resident	16 (50.0%)		
Staff	4 (12.5%)		
**US region of current institution**			
Midwest	11 (34.4%)	6 (37.5%)	5 (31.3%)
Northeast	5 (15.6%)	3 (18.8%)	2 (12.5%)
South	5 (15.6%)	0 (0.0%)	5 (31.3%)
West	11 (34.4%)	7 (43.8%)	4 (25.0%)
**Degree**			
PhD	29 (90.6%)	14 (87.5%)	15 (93.8%)
MS	3 (9.4%)	2 (12.5%)	1 (6.3%)
**Relationship status**			
Married or Living with Partner	23 (71.9%)	9 (56.3%)	14 (87.5%)
**Children**			
Yes	20 (62.5%)	5 (31.3%)	15 (93.8%)

To guide the interviews, a semi‐structured interview template was adapted from prior work studying academic physicians.^15^, ^16^, ^17^, ^18^, ^19^ The term “semi‐structured” here means that although the template provides a general framework, the interviewer will adapt the interview process to the participant based on the participant's own lived experience. The interview template was revised after critical review by an interdisciplinary working group with expertise in qualitative research in the healthcare setting. The interview questions were open‐ended and covered three primary topics: mentorship, work/life integration, and discrimination. We note that the interview guide was designed to explore additional topics beyond what is covered in the current report and that all transcripts were reviewed in their entirety to identify content related to resilience and well‐being. The complete interview guide is available in the appendix.

Qualitative analysis was performed on the de‐identified transcripts derived from the interviews. Thematic analysis^20^, ^21^ was used to develop the initial coding scheme which was subsequently improved and refined after 20% of the transcripts were reviewed. The purpose of thematic analysis is to identify and distill the important themes present across a dataset. All transcripts were qualitatively coded by at least two study team members, with differences resolved among the team by consensus discussion.

We used the following definitions within the qualitative analysis applied here: Resilience is “the process and outcome of successfully adapting to difficult or challenging life experiences, especially through mental, emotional, and behavioral flexibility and adjustment to external and internal demands”,^22^ burnout is a condition involving “physical, emotional, or mental exhaustion accompanied by decreased motivation, lowered performance, and negative attitudes toward oneself and others”,^23^ well‐being is “a state of happiness and contentment, with low levels of distress, overall good physical and mental health and outlook, or good quality of life”,^24^ and self‐efficacy is “the belief in one's capabilities to organize and execute the courses of action required to manage prospective situations”.^25^

We determined that thematic saturation (a point at which additional data do not yield more information relevant to the current study questions) was met after the analysis of the initial 32 interviews, and therefore did not extend the study beyond this initial group.^26^ For reference, a 2009 review paper of qualitative studies in influential journals of general medicine found that the median sample size for interview studies was 36 (range 9–383).^27^ Analysis was performed using MAXQDA (VERBI Software, 2017). Results are reported in accordance with Proposed Criteria for Systematic Evaluation of Qualitative Oncology Research by Hannum et al.^28^ To improve the readability of the interview quotations included in this manuscript, the quotations have been edited for clarity by removing filler words such as “um”, “so”, “like”, and “you know”.

## 3 RESULTS

### 3.1 Participant characteristics

Table 1 shows the demographic characteristics of the interview participants. In total, 24 unique CAMPEP‐accredited programs were represented in the study. Half of the participants were current MPRs, 37.5% (*n* = 12) were MP faculty, and 12.5% (*n* = 4) were MP staff. Of the residents who participated, the mean age was 33.8 +/– 5.5 years. Most had a PhD degree (*n* = 14, 87.5%), with the remainder holding MS degrees (*n* = 2, 12.5%). The majority were also married or living with a partner (*n* = 9, 56.3%), and 31.3% (*n* = 5) had children.

During the analysis of our semi‐structured interview data, we identified four themes that highlighted the importance of studying and addressing MPR burnout, resilience, and well‐being. The themes were: (1) the demanding nature of medical physics residencies, (2) the negative impacts of residency on MPRs during training and beyond, (3) strategies MPRs use to cope with residency stress, and (4) the role of professional societies in addressing residency‐related change. Select quotes from the interviews are shown in the text to illustrate each of the four themes. Additional supporting quotes are included in Table 2. Labels on the quotes throughout this manuscript indicate gender (W = woman, M = man) and role (R = resident, S = staff, F = faculty).

**TABLE 2:** Additional supporting quotes for each theme. Labels on the quotes indicate gender (W = woman, M = man) and role (R = resident, S = staff, F = faculty)

**Theme**	**Representative quote**
**Theme 1: the demanding nature of medical physics residencies**	*The match was the lowest point because most of my professors were very confident that with my Ph.D., with my academics, just by knowing me and having had me in their class and just interacting with me, they were very confident that I would have no problem with the match, but it was the exact opposite. When the match results came in and I didn't have any matches, that was very, very, very low because now I have to find a way to continue on the career, keep the momentum going, and there was a lot of difficulty before I landed this residency. So absolutely, the lowest, lowest point was the match because of the difficulty there. (MR)* *…there was a lot of worry in terms of “will I match to a residency program?” because there's a 65% match rate for people who are applying with these things. So, it's not a sure thing by any means. (MR)* *… the nature of our work is very demanding, at least in the way our job is laid out right now. It's a very demanding field, and it's unpredictable. There are those times when you think you're going to go home at 5:00 PM and things happen that you just can't predict, and you end up working until 10:00 or 11:00 at night. (MR)* *… People really praise other people that work weekends and long hours and things like that, and so I feel like that's kind of the mentality. … In [State,] I would stay until 10:00 or 11:00 doing patient‐specific QA 2–3 times a week and be expected to be there at 7:30 AM the next day. (WF)* *We followed the medical resident paradigm. … It's a situation that's prone to abuse as far as overburdening them with work. (MF)* *… I don't feel there's anything I can do as a resident, just being in the power situation that I am to change anything. Any time I do bring it up, the response I get is, “Oh, that's residency. Oh, I had to do that when I was a resident.” (WR)* *Last year, I had a point where I was in the clinic for 32 days in a row, and that wasn't anyone saying, “Hey, you need to be here every day for 32 days in a row.” That was just me feeling compelled to take care of the things I needed to take care of. (WR)* *That's the only negative about our particular residency. We don't have much PTO, no sick days or anything, especially compared to some residencies. Since last June, I took almost no time off. (WR)*
**Theme 2: the negative impacts of residency on medical physics residents during training and beyond**	*My girlfriend told me on a few occasions that she doesn't know anybody that works as much as me. I think that's a clear indicator that . . . it affects my relationships, or at least one of them specifically. (MR)* *…I am concerned that as a faculty physicist, a similar trend carries over where you are not encouraged to leave at an appropriate hour … You're asked to do things on the weekends. I don't know how much of that happens when you become faculty, but it is a concern that that might be the trend, and I also am considering a job that might be outside of medical physics because of that. (WR)* *Work‐life balance . . . I don't know what to advise them. They're pretty busy. I cannot tell junior physicists to work less. If he starts working less, he won't succeed initially. (MS)*
**Theme 3: strategies medical physics residents use to cope with residency‐related stress**	*I just accept it and then say, “Okay, well.” The number one thing that I have to focus on right now is training and getting that experience. … It gets easier with experience, I think. … It's a temporary situation, but you just have to accept it and then move on from there. (WR)* *Right now, it's a temporary thing while I'm doing this clinical training, but I don't want that to be a career long‐lasting thing where I'm always working, I'm always working. … So, we're fine with this temporary thing as a step to getting to the next phase of my career and phase of our life, but it's also something that comes into right now when I'm looking for my next position, it's one of the things that I try and detect while I'm going around interviewing, seeing, “Okay. What is your work‐life balance like?” because I don't want to be stuck somewhere where I'm overworked. (MR)* *I think one of the things that's helped me a lot has been maintaining friendship and social relationships, both within work and outside of work. I get along with a lot of the people really well in my residency, and we find times to go out and spend time socially outside of work. (MR)*
**Theme 4: the role of professional societies in addressing residency‐related change**	*So many of those residents are working far, far longer than I am. They're coming in on weekends. They don't get very many days off, things of that nature. It's something that people often actually joke about a lot in the field. They say that “a physicist has a good work‐life balance. It's because they have a resident to do this stuff late at night or early in the morning.” I don't think that that's really appropriately handled by the national organizations. I think there's a high expectation for some residency programs or some physics programs where a physicist just spends an incredible amount of time doing the job and not [doesn't have] much time left at home. (MR)* *I think part of that has to do with the fact that this residency for medical physicists is kind of a new thing. … My impression is that there's not a super well‐defined two years for what it means to go through medical physics residency, as opposed to a physician's residency where it's pretty tried and true and gets refined over the years, but there's very well outlined structure to it and I think a little bit more detail in terms of what they're expected to know and what they do throughout their residency to make sure they're learning these things. I get the impression that it's much more variable for medical physicists. (MR)* *Some of these institutions are charging $40,000 or $50,000/year for tuition and all of that business, and these people are graduating with an insane amount of debt, and they can't get into a residency program to do what the institution told them they'd be able to do with their degree. I think we really need to take a hard look at what the future of the field is, how many medical physicists do we need, and can we somehow match our number of graduates to that number of medical physicists? (MR)*

### 3.2 Theme 1: the demanding nature of medical physics residencies

One of the first major hurdles of residency is obtaining one of these coveted positions. Approximately 70%^29^ of the CAMPEP‐approved residencies participate in the annual MedPhys Match program, administered by National Matching Services, Inc. Some interview participants noted that the process of going through the match was a low point, or the biggest challenge, in their career thus far.

Biggest challenge . . . I think maybe getting into a residency program. In order to get board certification in our field, we have to graduate from specially accredited residency programs, and it's very, very competitive to get into those programs. There are more graduates from graduate programs than there are residency positions available. So that was really stressful to try and figure out how to secure a residency position, because if you can't get a residency position, then you can't take the boards, and then you can't continue in the field. So, I think that was probably the hardest part, at least so far with this job. (WR)

Many cited the low matching rates^30^ as a particular source of concern. These rates have varied from a low in 2015 of 39% to a high in 2019 of 63%. In 2021, the most recent year for which data are available, 130 of 218 applicants (60%) were successfully matched into a residency program.

I would say the biggest challenge was getting a residency. I mean, it's kind of a broken system right now to where the match rate is so low for people trying to get into residency that you have half of the people every year that spend all this time and financial resources into getting an upper‐level graduate degree to then be told, “Sorry. There aren't enough residency positions for everyone. You'll have to try something else.” I think that's a huge challenge. (MR)

Residents described feelings of burnout, whether or not the term was used explicitly. The language used by residents indicated a lack of self‐efficacy.^25^ Study participants used words and phrases such as “overwhelming,” “discouraging,” “demanding,” “crazy,” and “a rough time in your life.”

I would say that this residency is pretty much my life… that there probably isn't a lot to integrate beyond that working. … I think that work limits my ability to have any life. … I realize I am in residency. I'm trying to take that with a grain of salt. For whatever reason, it's designed to be overwhelming. (WR)

I think it's gotten harder because it's just continuous. I just feel like it's continuously knocking me down. … If I got a paper rejected, I could be like, “Okay, well I've gotten one accepted,” but here I just feel like every negative feedback I get is just compounding onto one another… There's nothing that I can be like, “Okay, well I did this right.” … It just feels like it's just continuously getting harder and going deeper and digging myself into a hole. Things that I'm trying to … I haven't figured out how to make things better. (WR)

Respondents discussed how job rank plays a role in a person's ability to structure their time and work demands in a way that best suits their goals, priorities, and personal wellness. MPRs tend to be at or near the bottom of the hierarchy within a typical clinical department.

As a junior member of the team, you sort of feel like you need to do more and you're not doing enough. I definitely have gotten burned out a couple of times, especially during residency, just trying to juggle the clinical load with the research load and any extra expectations or projects on top of that. (WR)

### 3.3 Theme 2: the negative impacts of residency on MPRs during training and beyond

With the current residency matching system in medical physics and the difficulty associated with achieving a residency position, family members may be separated from each other during training. This lack of control negatively impacts residents’ self‐efficacy, as also described in Theme 1.

I have a husband, and I'm doing a residency here, dividing the family right now. My husband and my son are in another state. I'm in this state, but I know that if this opportunity goes away, and so off goes my family. We're really suffering through this year of my residency, but if I don't do it, my career is going to be affected. (WR)

Long working hours are a standard in residency programs and impact both a resident's well‐being and their ability to adequately perform job duties.

I know a lot of medical residents are in the same situation where it's just like, Oh everybody just knows that as a resident you're working crazy amounts and crazy hours, but it's really bad and unsafe because we're dealing directly with patient care. If I'm working 18 straight hours, I'm not being a good physicist, and there's no way I can be a safe physicist. (WR)

Finally, burnout among residents may have negative consequences for medical physics as a field because of the inability to attract and retain a diverse and talented medical physics workforce.

There have been days where it's been just so hard, and I think, “What on earth am I doing? Why did I choose this?” There have been days where I've … not too seriously, but somewhat seriously considered just being done… (WR)

As residents transition to staff/faculty positions, they may carry this demonstrated lack of self‐efficacy with them. Alternatively, some physicists felt more control over their work/life balance and a sense of power to change elements that were not working for them. The ability to do this effectively depended on family and institutional support. The faculty also acknowledged that residents (and junior faculty) are burdened with the most difficult schedules.

I don't think that's a deliberate thing, but it's certainly easier to say “no” when you're partway up the food chain than when you're just starting out and you're at the beginning of the ladder and they ask you to do something or stay late at night, and you just say “okay.” And there's a tendency, I think, among the management or leadership to ask the junior faulty to take the late shift, to come in late at night, come in on the weekend. They get dealt the crappy hand more often than the more senior people. That may be detrimental to the field overall, that we might burn people out early on, abusing them because we can. (MF)

### 3.4 Theme 3: strategies MPRs use to cope with residency‐related stress

Institutional and field‐wide changes to improve the well‐being of MPs will require significant time and resources. For now, many residents are finding their own ways to manage this stress. Some residents focused on the time‐limited nature of training.

My wife and kids and I all understand that this is a residency. 1) Residencies are temporary, but, 2) they're usually pretty demanding. I think we're all on the same page that right now we're going to have to sacrifice a lot of time together, but in the end, it will kind of even out… (MR)

Residents expressed hope for the future: that their working hours and responsibilities would become more manageable after residency.

Currently, as a resident, my hours especially at the beginning were pretty terrible. I was working probably 70 h a week, something like that on average. … I'm hoping that once I transition to the faculty, it sounds like the hours should also go back down to normal, and I should also be working closer to 50/week… (WR)

One resident focused on how residency helps them toward their goal of taking care of their family.

It does get stressful and hard sometimes. So, I need a reminder that I'm also doing this for my family, that it's not just a personal thing that I want to do. It's something I want to do to help take care of my family. Whenever I can be around them, it helps me too. You know, motivation, keep going, push through hard times, it'll get better. Hopefully, you know, it will be enough to take care and support them, to help them have a good life and provide for their needs and things like that. (MR)

Residents noted the importance of getting support from fellow residents/colleagues.

All the residents sit together, and so we all talk amongst ourselves. … It's good because we're all going through a similar thing. So, we make jokes about, “Oh, this is ridiculous,” but that joking… It sort of helps relieve some of that “this is a rough time in your life, but we'll get through it all together.” So having them as a support group is great. (WR)

Others discussed working on their personal mindset.

I just have to learn to constantly prioritize and re‐prioritize things and just let some things go. … But for now, it's just trying to keep calm and not let things get to me and then just letting go of some things that aren't important. (WR)

One resident made it a long‐term goal to get a better work‐life balance while still working in academic medicine.

[My goal is] probably still staying at an academic center, but maybe trying to ease up and cut back on some of my hours so I can have more time with family. That might be nice. (WR)

Time away from the clinic was noted to be important for resident well‐being, however, ongoing clinical commitments often make this difficult for trainees to arrange.

I felt this more as I progressed through residency, … that the only way I can get enough personal time to feel like I'm in touch with my life outside of work is to take a vacation. (MR)

### 3.5 Theme 4: the role of professional societies in addressing residency‐related change

Respondents articulated a need for more resources and support for residents. Professional societies were perceived as able to help improve the current culture around MP well‐being for the benefit of the field.

I think the residents are probably the ones who are most likely to be handed a schedule that is not a great work‐life balance. There's not much in place to stop that from happening. … people being overburdened and having a poor balance and maybe burnout would be for residents. I'm not currently aware of anything in place to try to help them or try to regulate… that those schedules are not overburdening. Also, they're least likely to be able to speak up for themselves, just because they're in this training position. … I would say the only one that I would suggest would be if … a group like CAMPEP or somebody could have some sort of a requirement for residents. That's where I see is the weak spot, is how residents are treated. (MF)

Resident‐focused resources and policies, along with clearly defined residency guidelines, would also be valued.

I mean it would be nice if there was some kind of awareness within the communities about how to maintain or even change some of the cultures. It's not such a problem in diagnostic, but I know therapy physicists working really long hours, I would imagine that would be challenging. It would be nice if that wasn't the culture. (WR)

There may also be a potential for professional organizations to further analyze workforce issues and make recommendations.

[Residency is] kind of a bottleneck in our field … if you don't get a residency, your career options are severely limited beyond that. (MR)

## 4 DISCUSSION

In this study, we have qualitatively investigated MPR well‐being and resilience, the potential impacts of each, and suggested potential interventions to improve them. Resiliency, of which self‐efficacy is one component, acts as a protective factor against burnout.^31^, ^32^, ^33^ There is evidence that healthcare workers are overall more resilient than the general employed US population, but even so, burnout rates remain seriously high.^34^

### 4.1 The demanding nature of medical physics residencies

A residency in medical physics has several qualities that may lead to burnout. These include difficulty in obtaining a residency position, long, unpredictable, and unusual hours, a considerable amount of didactic material to learn, limited control over personal schedules, separation from families, busy environments, the psychological impact of patient care responsibilities (for which MPRs often receive no formal training), a requirement for teamwork across multiple professional groups, and uncertainty about the future.

Within our study cohort, even the process of obtaining a residency was noted to be highly stressful. Women and other excluded groups (e.g. racial/ethnic minoritized populations, sexual and gender minorities, disabled populations, and others) face additional challenges and discrimination that compound these effects.^14^, ^35^, ^36^ Most residency programs currently participate in the MedPhys Match program, administered by National Matching Services Inc., with the match rate hovering around 60% over the past three years. In 2020, the average number of positions ranked per matched applicant was 8.9 (prior to the coronavirus disease 2019 [COVID‐19] pandemic when interviews were primarily conducted in‐person), illustrating some of the expense and stress, both for applicants and programs, associated with the residency interview period.^37^ A potential solution for the field of medical physics could involve adapting the proposed optional early result acceptance program for physicians, where residents are allowed to apply to a limited number of programs (five in the proposed strategy), and programs can fill half of their available slots from this cohort.^38^

### 4.2 The negative impacts of residency on MPRs during training and beyond

Burnout is associated with increased rates of depression, substance abuse, poor relationships with family and co‐workers, medical errors, and suicidal ideation.^34^, ^39^ Long working hours are a driver of burnout and have measurable impacts on health and wellbeing among medical professionals.^40^, ^41^ A recent study by the World Health Organization found that exposure to “long working hours” (defined as working more than 55 h/week), significantly increased the risk of heart disease and stroke.^13^ For comparison, although these data are not broken out by job role, the 2020 AAPM Professional Survey indicates that 17% of physicists working in medical school or university hospital settings are working 55 h or more per week.^42^ The Accreditation Council for Graduate Medical Education (ACGME) limits resident working hours to 80 per week, averaged over a 4‐week period.^43^ Some medical physics residencies follow this guidance, while others do not have specific policies in place.^44^, ^45^, ^46^

As described by some participants in this study, low levels of MPR well‐being may also lead to attrition. This has been demonstrated in many other areas of medicine,^47^, ^48^, ^49^ leading to extraordinarily high costs to the healthcare system,^50^ and may even result in an expanding gender gap in the field,^51^ especially in the wake of the COVID‐19 pandemic.^52^

### 4.3 Strategies MPRs use to cope with residency stress

MPRs can be trained (or self‐train) in personal resilience and wellness strategies. Many medical residencies now have resident‐led wellness initiatives in place that could be mirrored in the physics arena. A 2016 systematic review of well‐being in residency by Raj found that autonomy, the building of competence, and strong social relatedness were important factors, in addition to sleep and time away from work.^53^

As described by some participants, peer support is a critical component of resident resilience and well‐being.^4^, ^54^, ^55^ The AAPM Students and Trainees Subcommittee provides an important connection for residents to interact with and support one another, as well as a link between residents and their primary professional society. The authors of this study strongly encourage that medical physics residency program directors provide a forum for residents to freely discuss wellness‐related issues, both personal and professional, and to promote an open dialogue about resident concerns.

### 4.4 The role of professional societies in addressing residency‐related change

Ultimately, to remedy burnout, the structural contributors must be addressed by those with the influence to encourage the wholesale cultural transformation that is necessary to improve MPR wellness. AAPM, the Society of Directors of Academic Medical Physics Programs (SDAMPP), and CAMPEP can and do meaningfully contribute to a culture that combats resident burnout to promote the health of the field, with several initiatives already in place. SDAMPP started and maintains a residency interview calendar to help make the interviewing process more straightforward. The Medical Physics Residency Training and Promotion Subcommittee of AAPM is discussing developing online modules for professional competencies such as communication and teamwork, discussing methods and metrics to evaluate medical physics residency programs (Work Group on Entrustable Professional Activities for Medical Physics), and connecting residents across programs together in a multi‐institutional journal club (Work Group on Multi‐Institutional Journal Clubs for Residency Programs). AAPM regularly assesses working hours in the association's annual Professional Survey (https://www.aapm.org/pubs/surveys.asp), and in the past has sponsored a workforce study that includes residents (https://www.aapm.org/pubs/protected_files/surveys/workforce/Synthesis.pdf). The AAPM Diagnostic Workforce Subcommittee is working on modeling staffing needs using projection models from the 2020 AAPM Imaging Physics (Diagnostic) Workforce Survey. The AAPM Spring Clinical and Annual Meetings have included sessions related to well‐being that are available to members on the AAPM Virtual Library (https://www.aapm.org/education/VL/). Additionally, the President's Symposium “Building Bridges” at the 2019 AAPM Annual Meeting described some of the many benefits of diversity in medical physics and generational differences in work/life integration strategies and expectations. Finally, AAPM provides many mentorship opportunities for students and junior physicists, including the Science Council Associates Mentorship Program, Diversity Recruitment through Education and Mentoring, the Summer Undergraduate Fellowship and Outreach Program, and the Professional Council Mentorship program. AAPM has initiated the Volunteer Engagement Program, a new committee membership classification for early‐career MPs interested in becoming involved in volunteer efforts. Connections across different generations of MPs have the potential to improve wellness and resilience in the field as a whole. We note that the above list is only a sampling of the available programs and not a comprehensive catalog of resources.

The results of the current study suggest new ideas for strategies to tackle burnout in trainees. Both residents and faculty/staff participants in our study expressed interest in further guidance on reasonable working hours and staffing requirements. The importance of mentorship in healthcare has been well‐studied.^56^ In order to help trainees navigate the difficulties described here during training and beyond, AAPM could provide further opportunities for structured mentorship, especially with respect to professional and personal development. Presentations at society‐sponsored meetings should continue to highlight wellness‐related initiatives and provide a platform for open, constructive discussion about these and other related topics.

Professional society‐endorsed recommendations about medical physics residency training programs^2^ should be expanded to address measures to protect and encourage resident well‐being. There is also precedent in the field of medicine for accrediting bodies to require and verify resident well‐being in their accredited programs. For example, the ACGME includes core program requirements that focus on resident well‐being and conducts an annual survey for all medical residents and fellows in accredited programs to assess whether programs are following working hour restrictions, fostering an inclusive work environment, providing access to mental health counseling, offering safe transitions of care when residents are fatigued, and ensuring the professionalism of supervisors, among other key measures.^57^ These data are then made available during reviews of accreditation for individual programs and their sponsoring institutions. Programs are required to maintain a 70% response rate to avoid additional review. This, or a similar type of strategy, could be adopted by CAMPEP to ensure the well‐being of residents in accredited medical physics residency programs, and to allow an avenue for residents to confidentially report malignant programs. Maintaining a 70% response rate may be more challenging for MP because these programs are typically smaller; for example, if there are only two or three residents in a program, all residents would have to respond in order to meet the 70% threshold.

A limitation of this study is the small cohort size used to conduct this qualitative research. Because of this, here we have considered all residents as a single cohort and have not been able to explore how excluded groups who are underrepresented in Science, Technology, Engineering, Math, and Medicine may be further impacted by the concerns described here. We also acknowledge that there may be participation bias, where participants with strong opinions on the topics of mentorship, work/life integration, and discrimination may have been more likely to respond to our invitation. Nevertheless, the quantity of rich textual data obtained and the rigorous approach to analysis ensure that valuable insights have been captured. In order to help mitigate the recognized limitations, in a follow‐up phase of this study, this qualitative analysis will be used to inform a quantitative survey instrument that will be distributed to a diverse group of MPs in order to test the generalizability of the study findings and to broaden our knowledge about the prevalence of burnout and the current state of residencies in the field of medical physics.

## 5 CONCLUSIONS

Residency is a required step in the process of becoming a board‐certified MP. The highly talented and committed individuals in these programs represent the future of the field of medical physics. Professional societies, institutions, faculty and staff MPs, and residents themselves should recognize the challenges inherent to this period of intensive training and consider policy initiatives similar to those implemented in medical residencies to support and promote the well‐being of the future of our field.

## CONFLICT OF INTEREST

Jean M Moran reports grants from NIH, grants from Blue Cross Blue Shield of Michigan, grants from Varian Medical Systems, outside the submitted work. Joann I Prisciandaro reports other from Varian Medical Systems Inc., non‐financial support from C4 Imaging LLC, outside the submitted work. Reshma Jagsi reports stock options as compensation for her advisory board role in Equity Quotient, a company that evaluates culture in health care companies; she has received personal fees from Amgen and Vizient and grants for unrelated work from the National Institutes of Health, the Doris Duke Foundation, the Greenwall Foundation, the Komen Foundation, and Blue Cross Blue Shield of Michigan for the Michigan Radiation Oncology Quality Consortium. Kelly C Paradis, Kerry A Ryan, Spencer Schmid, Anna Laucis, Christina H Chapman, Terri Bott‐Kothari, Samantha Simiele, James M Balter, Martha M Matuszak, and Vrinda Narayana, have nothing to disclose.

## AUTHOR CONTRIBUTIONS

All authors contributed to the conception or design of the work or the acquisition, analysis, or interpretation of data for the work. All authors contributed to drafting the work or revising it critically for important intellectual content. All authors gave final approval of the version to be published.

## Supporting information



SUPPORTING INFORMATION
