# Supplementary material for: A qualitative investigation of resilience and well‐being among medical physics residents
Source: J Appl Clin Med Phys. 2022 Feb 7;23(3):e13554. doi: 10.1002/acm2.13554 (PMC8906227; doi:10.1002/acm2.13554)
Supplement: Supplementary file 1 — SUPPORTING INFORMATION [file ACM2-23-e13554-s001.docx]

**Current Status**

I’ll start by asking some basic questions about your career so far and how you spend your time.

In what field is your degree?

Briefly, what drew you to a career in medical physics?

Can you briefly tell me about what your day to day job entails and how you distribute your time between various activities, such as clinical work, research, and teaching?

**Mentorship**

I’d like to understand a little bit more about how your career developed and if mentoring or other relationships and resources played a role.

Do you or have you had any mentors in your career and if so, can you briefly tell me about those relationships?

**If yes, these are probing questions to ask if not previously answered:**

What kinds of roles have mentors played for you?

How would you say that the roles of your mentors have changed over time?

How did you come to develop your relationships with your mentors?

Do you think this was similar to the way your colleagues developed mentorship relationships?

How active do you feel you had to be in seeking out these relationships?

Could you tell me about a time when a mentor really helped you out?

Is there a specific time you could tell me about when you wished you had a mentor who could help you?

What do you think makes mentoring relationships work well?

What do you think makes mentoring relationships work poorly?

Do you think that mentorship relationships are affected by the gender or racial identities of the mentee and mentor? If so, in what ways?

How important are mentorship relationships to achieving success in the field of medical physics? Why?

**Work/life integration**

Now we’ll talk about how you integrate your home life with your work life.

Would you say that family or other personal demands affected your career or your ability to succeed?

Do you currently have concerns about the integration of your work and personal or family life?

**If yes, and if not already answered:**

Can you tell me about these concerns?

What have you done to try to resolve these concerns?

Can you tell me about a time when these concerns might have been especially troublesome for you?

Are you currently married or living with a partner?

**If yes:**

Does your partner work? What kind of job does he or she have?

Would you say that both your careers take equal priority in your family, or does one career tend to take priority?

In your family, how do you split up responsibilities in the home?

Do you have children?

**If yes, and if not already answered:**

How old are they?

Are they still living at home?

**If yes,**

Where are they during the day?

In your family, how do you split up child care responsibilities?

Did you consider altering your career goals once you had children?

**If yes, and if not already answered:**

How did you decide what to do?

Did your feelings about this change over time?

Are there any particular strategies that you use to avoid burnout at work or at home?

Is there anything you’d like to add about the way you balance your personal life and work life or avoid burnout that we haven’t covered?

Would you say professional societies in the field of medical physics do enough to encourage and support healthy work/life integration?

Is there anything that you would like to see medical physics professional societies do to support healthy work/life integration?

**Discrimination**

Okay, I now have a few questions regarding discrimination that you may have experienced in your career.

How well do you feel you fit in at your institution?

Do you ever feel excluded at your institution?

Do you ever feel excluded by colleagues at other institutions?

From what you’ve seen in your career, do you think gender impacts the way a medical physicist might be treated by his or her colleagues? If so, in what way?

(**If not already answered**) Would you say that this has changed over time?

Do you think gender affects a person’s ability to succeed in a career in medical physics? If so, in what way?

Do you feel that your gender or any other personal attribute has affected your career or your ability to succeed?

Have you ever personally experienced harassment (during your career) that was related to your gender or other personal attribute?

**If yes:**

Would you be willing to share what happened?

What was your response to these experiences?

**Personal goals**

Lastly, we’ll talk a little bit about your personal challenges and goals.

Can you tell me about a time in your career when things were at a high point?

What about a low point?

Would you be willing to share a story about a time when you experienced rejection, including what happened and how you responded?

What would you say has been the biggest challenge in your career so far?

At this point in your career, how satisfied are you with the way things have turned out?

Thinking about the next 5 years, can you list a few of your primary goals in the shorter term?

Thinking about the next 10 – 20 years, can you list a few of your primary goals in the long term?

**Other thoughts / reflections (if time permits)**

Well, we’re nearing the end of our interview today, and I’d like to take some time now to ask you for some final thoughts on what we’ve talked about.

Specifically, if you could give one recommendation or piece of advice to medical physicists who are just starting out in their careers or are still in training, what would it be?

Do you have any ideas for policies or institutional practices that might be helpful to those physicists?

Is there anything that professional societies could do to help?

Finally, is there anything you’d like to add that we haven’t had a chance to talk about?
